# Toll-8/Tollo Negatively Regulates Antimicrobial Response in the *Drosophila* Respiratory Epithelium

**DOI:** 10.1371/journal.ppat.1002319

**Published:** 2011-10-13

**Authors:** Idir Akhouayri, Claire Turc, Julien Royet, Bernard Charroux

**Affiliations:** IBDML, UMR 6216 CNRS, Université Aix-Marseille, Marseille, France; Stanford University, United States of America

## Abstract

Barrier epithelia that are persistently exposed to microbes have evolved potent immune tools to eliminate such pathogens. If mechanisms that control *Drosophila* systemic responses are well-characterized, the epithelial immune responses remain poorly understood. Here, we performed a genetic dissection of the cascades activated during the immune response of the *Drosophila* airway epithelium *i.e.* trachea. We present evidence that bacteria induced-antimicrobial peptide (AMP) production in the trachea is controlled by two signalling cascades. AMP gene transcription is activated by the inducible IMD pathway that acts non-cell autonomously in trachea. This IMD-dependent AMP activation is antagonized by a constitutively active signalling module involving the receptor Toll-8/Tollo, the ligand Spätzle2/DNT1 and Ect-4, the *Drosophila* ortholog of the human Sterile alpha and HEAT/ARMadillo motif (SARM). Our data show that, in addition to Toll-1 whose function is essential during the systemic immune response, *Drosophila* relies on another Toll family member to control the immune response in the respiratory epithelium.

## Author Summary

Invertebrates solely rely on innate immune responses for defense against microbial infections. Taking advantage of its powerful genetics, the fly *Drosophila melanogaster* has been extensively used as a model system to dissect the molecular mechanisms that control innate immunity. This work led to the discovery of the essential role of the Toll-1 receptor in triggering the systemic immune response in flies, and paved the way for the discovery of the function of members of the Toll-like receptor (TLR) family in mammalian immunity. Whereas all TLRs are implicated in the mammalian immune response, Toll-1 was, so far, the only *Drosophila* Toll family member to be involved in the regulation of the immune response. In the present study, we show that another Toll family member, Toll-8 (Tollo), plays an important role in controlling the respiratory epithelium immune response. Our data indicate that, by antagonizing the IMD pathway, Tollo is preventing over-activation of the antibacterial response in the airway epithelium.

## Introduction

Although the innate immune system is a primitive host defense mechanism, it involves a sophisticated repertoire of humoral and cellular responses both acting systemically and locally [1]. In recent years, the *Drosophila* model organism has proven to be an invaluable system in dissecting in great details the genetics and cellular mechanisms regulating the innate immunity [2]–[3]. One fundamental mechanism common to humans and *Drosophila* immunity involves signaling by receptors of the Toll family. Upon microbial infection, human TLRs activate the synthesis of cytokines and other regulatory molecules that stimulate the adaptive immune system [4]. In *Drosophila*, Toll signalling leads to the activation of the systemic immune response, which is characterized by the synthesis of AMPs by the fat body cells [5]. Upon secretion into the circulating blood, these AMPs provide systemic protection against fungi and bacteria. In the mammalian innate immune response, bacteria are directly sensed by TLRs [6]. In contrast, these microorganisms are detected in *Drosophila* by another class of proteins, the Peptidoglycan Recognition Proteins (PGRPs), also present in the human proteome [7]–[9]. Recognition of Lys-type peptidoglycan (PGN) (mainly found in Gram-positive bacteria cell wall) by the circulating PGRP-SA protein triggers a protease cascade involving successively Mod-Sp, Grass, and the Spätzle-Processing-Enzyme (SPE) [8], [10]–[11]. Upon activation, SPE becomes competent to transform the zymogen pro-Spätzle into an active ligand for the Toll receptor, inducing its dimerization and intracellular signalling [12]. Production of AMPs after infection by Gram-negative bacteria, is however largely independent of the Toll pathway but rather relies on another NF-κB signalling cascade named IMD [13]. Sensing of Gram-negative bacteria upstream of the IMD pathway takes place at the plasma membrane, through PGN recognition by the transmembrane PGRP-LC receptor. Binding of DAP-type PGN (present in Gram-negative bacterial cell wall) to PGRP-LC induces its dimerization, which, in turn, triggers IMD-dependent intracellular events enabling the nuclear translocation of the NF-κB transcription factor Relish [14]–[17].

Although penetration of infectious microbes into the body cavity, and consequently, activation of a systemic immune response, is a rare event, interactions between microbes and epithelia take place constantly throughout the life of all metazoan. This implies that these barrier epithelia must be armed with efficient systems for microbial detection and elimination. However, these epithelia that act as interfaces with the external environment, share some characteristics that could be seen as detrimental for the needs of an effective immune system. Indeed, they usually have large surface areas and consist of thin structural layers, thus representing ideal entry points for pathogens. In this respect, the airway epithelium is unique among all epithelia, since it has a very delicate structure and is constantly exposed to a plethora of airborne pathogens. This could explain the occurrence of a great variety of inflammatory lung diseases, including asthma, chronic obstructive pulmonary disease or cystic fibrosis [18]–[19]. Elucidation of the primary steps that lead to chronic inflammation of the mammalian lung is obstructed by the complexity of inflammatory responses in this organ. Animals with a much simpler organization, such as the fruit fly, might help us clarify the basic architecture of this epithelial immune response, thereby helping to unravel the mechanisms that lead to chronic inflammation of the airways. Although of much simpler organization, the fly's airway system shows striking similarities with the mammalian lung regarding both its architecture and its physiology [20]–[25].

In this report, we present a detailed description of the mechanisms that regulate AMP production in the *Drosophila* respiratory epithelium. We show that, in contrast to systemic fat body immune response, the IMD pathway can be activated non-cell autonomously in the tracheal network. We present evidence that IMD pathway activation is tightly regulated in the cells of the respiratory epithelium. We demonstrate that the molecular mechanisms underlying IMD down-regulation following infection, are different from those previously reported in the gut and in the fat body, and rely on a dialog between two antagonist pathways. The production of AMPs in the trachea is positively regulated by the IMD pathway, which is counterbalanced by a negative regulation from a signalling cassette, whose upstream receptor is a member of the Toll family, Toll-8/Tollo. Our data suggest that the Spz2/DNT1 cytokine is a putative Tollo ligand in this process, and that Ect4, the *Drosophila* ortholog of the human Toll/Interleukin-1 Receptor (TIR) domain-containing protein SARM mediates Tollo signalling during tracheal immune response.

## Results

### AMP activation followed a stereotypical pattern in the Drosophila tracheal network

**Figure 1:**
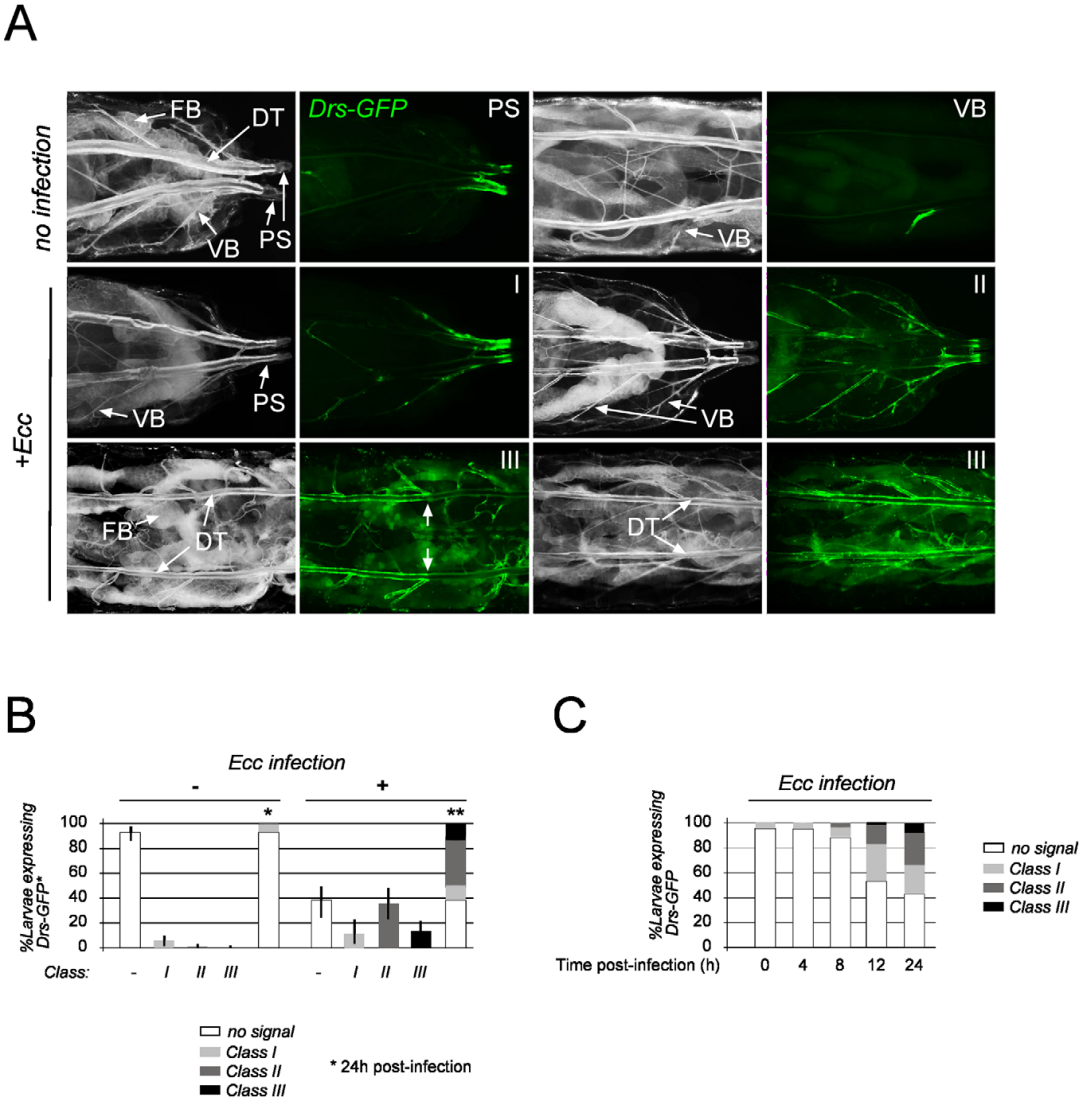
Spatio-temporal expression of tracheal *Drosomycin-GFP*. (A) Dorsal view of *Drs-GFP* third instar larvae. In non-infected larvae, sporadic GFP expression is visible in posterior spiracles (PS) and/or in visceral branches (VB). Upon *Ecc* infection, larvae display a highly reproducible pattern of GFP expression, classified as follow: Class I, PS + posterior VB only; Class II, PS + all VB; Class III, VB + dorsal trunk (DT), with *Drs-GFP* first in the anterior half (limit marked by arrows) and only later, in the entire trunk. FB: fat body. (B) Quantification of tracheal *Drs-GFP*-positive larvae and distribution of classes upon *Ecc* infection. Each histogram corresponds to the mean value of 5 experiments. A total number of 100 larvae were counted for each experiment. Statistics apply for the “no signal” and the class III categories only. Values indicated by identical symbols (* or **) are not significantly different (*P*>0.05) from one another. All other differences are statistically significant (*P*<0.05). (C) Quantification of tracheal *Drs-GFP*-positive larvae at 4h, 8h, 12h and 24h post-infection by *Ecc*. Each histogram corresponds to the mean value of 3 experiments. A total number of 90 larvae were counted for each experiment.

The tracheal system is a relatively simple model system that has provided an important insight into the biology of branching morphology [22]. It is a tubular structure covered by a lumenal cuticular lining that forms a physical barrier against dehydration and invading microorganisms [20]. This network consists of a monolayer epithelium made up of two dorsal trunks (DT) connected to several visceral branches (VB) bringing oxygen to internal tissues. Upon infection by the entomopathogenic bacterium *Erwinia carotovora carotovora* (*Ecc*), these epithelial cells produce a cocktail of AMPs including Drosomycin, Drosocin and Attacin [26]–[27]. In order to perform a detailed spatiotemporal analysis of this epithelial response, we used a *Drosomycin-GFP* reporter transgene (*Drs-GFP*) and monitored the response of this tissue after infection. When reared on conventional medium, a few larvae showed sporadic tracheal *Drs-GFP* expression mainly in VB, and rarely in the posterior part of the DT, namely the posterior spiracles (PS) (Figure 1A and 1B). This basal *Drs-GFP* tracheal expression was qualitatively and quantitatively similar in larvae reared conventionally or under axenic conditions (data not shown). Upon infection with *Ecc, Drs-GFP* activation followed a somewhat stereotypical pattern. Responding larvae were categorized into three classes according to their *Drs-GFP* expression pattern (Figure 1A and 1C), namely, larvae expressing GFP in PS and in posterior VB only (class I), in PS and all VB (class II) and in VB and DT (class III). Kinetic experiments showed that GFP signal was first detected in PS and then spread into VB (Figure 1A, C). The reporter was only later activated in the DT and, unexpectedly, first in the anterior half and then in the entire trunk (Figure 1A). Although we appreciate that the expression patterns of reporter transgenes can slightly deviate from those of the actual AMP, the *Drs-GFP* expression patterns observed cannot easily be attributed to a progressive diffusion of bacterial elicitors (such as PGN) from the spiracles into the tracheal network, but rather speak for more complicated mechanisms in tracheal AMP activation.

### IMD pathway activation is not strictly cell autonomous in the trachea

**Figure 2:**
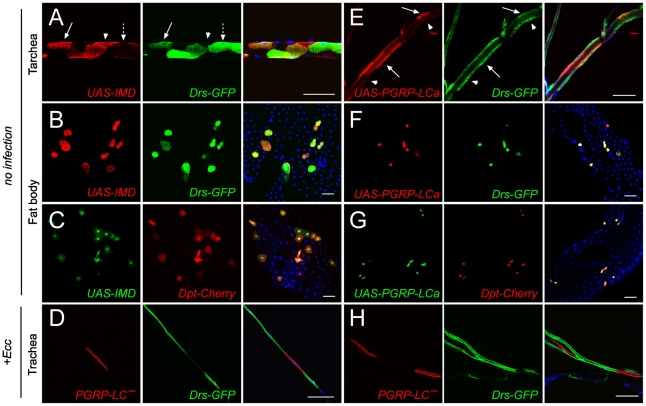
IMD pathway activation is not strictly cell-autonomous in trachea. IMD overexpressing clones in trachea (A) and fat body (B, C) cells are marked by RFP (A, B) or GFP (C) expression. (A) IMD overexpression activates *Drs-GFP* cell-autonomously (66%, n = 68) (arrow) and non-autonomously (12%, n = 68) (dashed arrow) in the trachea. Note that 34% (n = 68) of IMD-expressing cells are unable to activate *Drs-GFP* (arrow head). On the contrary, 98% (n = 38, for *Drs-GFP*) and 97% (n = 57, for *Dpt-Cherry*) of IMD-expressing cells activate *Drs-GFP* (B) *Dpt-Cherry* (C) in a strictly cell-autonomous fashion in the fat body. Clones overexpressing PGRP-LCa in trachea (E) and fat body (F, G) are marked by RFP (E, F) or GFP (G) expression. Tracheal cells expressing PGRP-LCa activate *Drs-GFP* autonomously (74%, n = 46) (arrow) and non-autonomously (39%, n = 46) (arrow head) (E). Fat body cells expressing PGRP-LCa (99%, n = 48 for *Drs-GFP*) and (98%, n = 63 for *Dpt-Cherry*) activate *Drs-GFP* (F) and *Dpt-Cherry* (G) autonomously. RFP expressing clones never activates Drs-GFP cell-autonomoulsy (0%, n = 52) nor cell non-autonomously (0%, n = 52) in the trachea. (D, H) Visceral branches of *Ecc-*infected larvae containing *PGRP-LC* mutant clones, marked by RFP expression (MARCM see Methods). Cells lacking *PGRP-LC* (red) are unable to activate *Drs-GFP* expression, while surrounding cells do (green). Nuclei are stained with Dapi (blue). Scale bar is 100 µm.

Next, we compared the mode of activation of AMP following forced immune pathway activation in the trachea and in another immune tissue, the fat body. Although activation of *Drosomycin* transcription is mainly controlled by the Toll pathway in the fat body (but can be activated by ectopic IMD pathway triggering, see later), it is strictly IMD-dependent in the trachea [5], [26], [28]. Indeed, over-activation of the IMD (*UAS-PGRP-LCa*, *UAS-IMD*), but not of the Toll pathway (*UAS-spz act*, *Tl^3^*), is sufficient to induce tracheal expression of *Drs-GFP* in non-infected larvae (Figure S1A and S1B). Concomitantly, loss-of-function mutations in IMD pathway components (Relish, PGRP-LC, IMD) prevent *Drs-GFP* tracheal activation in infected larvae, whereas Toll signalling mutants such as *spz* or *Dif* do show a wild-type tracheal response upon infection. To analyze whether all tracheal cells were competent to trigger AMP production upon IMD pathway activation, we induced UAS-IMD expressing clones in tracheal cells, using fat body clones as controls. Overexpression of IMD led to a strictly cell-autonomous and fully penetrant activation of both *Drs-GFP* and *Dipt-Cherry* in the fat body (Figure 2B and 2C). In the trachea, although most IMD-expressing cells showed *Drs-GFP* expression, a fraction did not (Figure 2A). In addition, *Drs-GFP* activation was not always associated with the expression of the UAS-IMD transgene (Figure 2A), suggesting that IMD pathway activation in trachea is not strictly cell autonomous. These results were confirmed by using a UAS-PGRP-LCa transgene that activated *Drs-GFP* both autonomously and non-autonomously in trachea cells (Figure 2E) but strictly cell-autonomously in the fat body (Figure 2F). We next addressed whether PGRP-LC function was required cell-autonomously for IMD pathway activation in the trachea upon infection. Analysis of MARCM loss-of-function clones for PGRP-LC indicates that tracheal cells mutant for PGRP-LC were totally impaired in their ability to trigger *Drs-GFP* expression, following *Ecc* infection (Figure 2D and H). These results indicate that, although PGRP-LC is essential in tracheal cells for IMD pathway triggering, IMD pathway activation in one tracheal cell can spread to neighboring cells. This contrasts with the strictly cell-autonomous IMD-dependent immune response observed in fat body cells.

### Tollo is expressed apically in the Drosophila trachea cells

**Figure 3:**
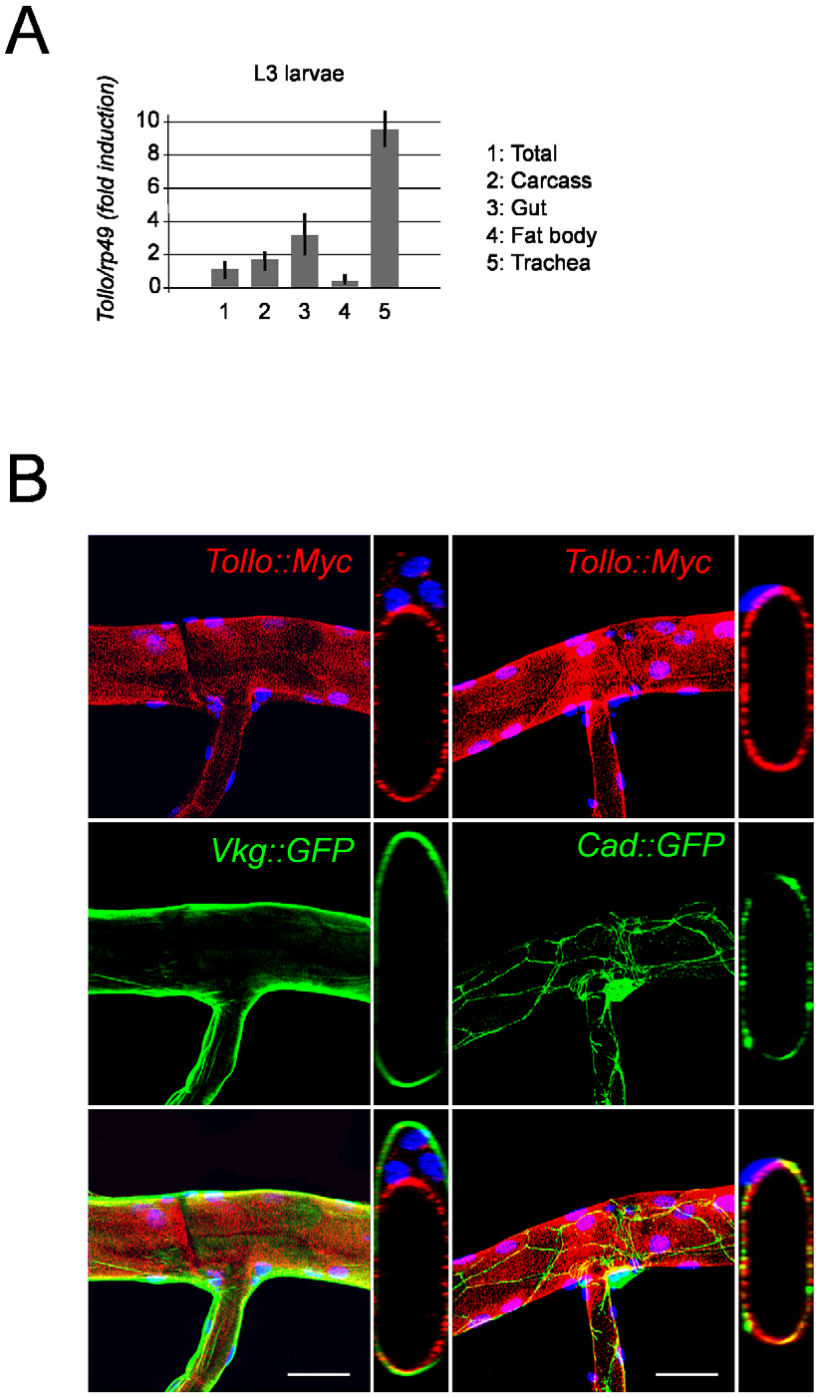
Apical localization of Tollo protein in tracheal cells. (A) *Tollo* mRNA levels detected by q-RT-QPCR in third instar larval tissues. Histograms correspond to the mean +/- SD of 3 experiments. *Tollo* expression in total larva extract was set at 1. (B) Larval trachea expressing a Tollo::Myc fusion detected with an anti-Myc antibody (red) and expressing the basal membrane fusion protein Viking::GFP (green, left panels) or the apical marker Cadherin::GFP fusion (green, right panels). Z sectioning shown in smaller panels indicate that Tollo::Myc co-localizes with the Cadherin::GFP fusion and is excluded from the basement membrane. Both confocal stacks (large panels) and Z sectioning (small panel) are shown. Nuclei are stained with Dapi (blue). Scale bar is 50 µm.

In order to get a further insight into the mechanisms that control AMP induction in trachea, we looked for putative immune genes expressed in this tissue. A recent report identified the repertoire of all immune genes expressed in the trachea [29]. One of the striking data of this study, [confirmed by FlyAtlas (http://flyatlas.org/)] was that, in addition to Toll itself, two other Toll family members, 18-Wheeler (Toll-2) and Tollo (Toll-8), are strongly expressed in trachea. 18-wheeler being implicated in developmental processes with indirect impacts the immune response [30], we focused our study on the putative function of the Tollo transmembrane protein in the tracheal immune response. Using Lac-Z reporter lines (data not shown) and q-RT-PCR (Figure 3A), we confirmed that *Tollo* mRNA is highly enriched in the tracheal epithelium, and expressed at lower levels in other tissues. To investigate the subcellular localization of the Tollo protein, we genetically associated a *UAS-Tollo::Myc* construct with the trachea-specific *Breathless-Gal4* driver (*Btl-Gal4*). Anti-Myc antibody staining suggested that Tollo was localized apically at the cell membrane facing the airway lumen (Figure 3B). Double staining experiments showed that Tollo::Myc partially co-localized with the apical marker Cadherin::GFP, but was mutually exclusive with Viking::GFP, a basal membrane-associated protein (Figure 3B). These results indicate that Tollo is a protein enriched in the tracheal epithelium with an apical subcellular localization.

### AMPs are specifically over-produced in infected Tollo mutant trachea

**Figure 4:**
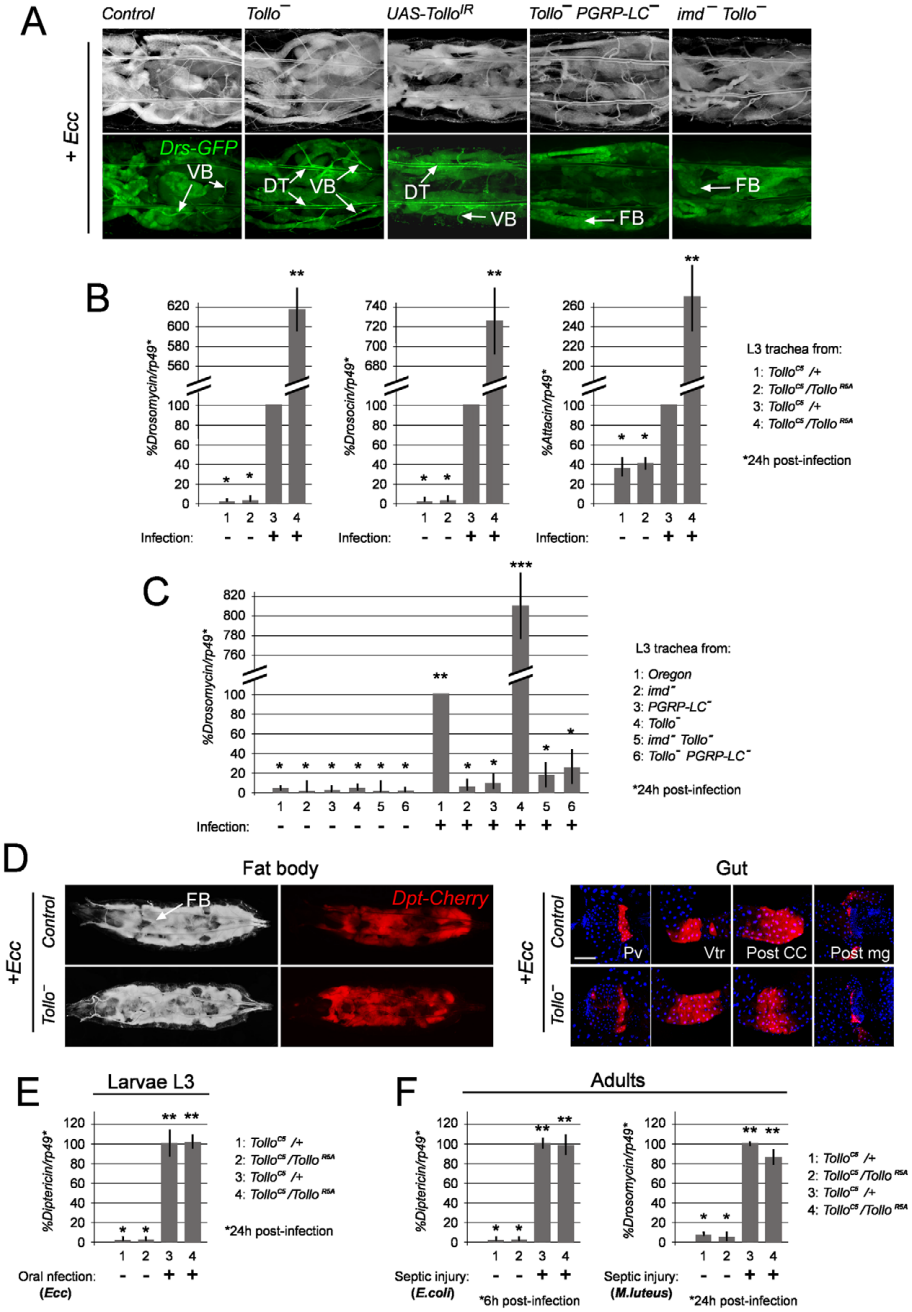
Negative regulation of tracheal immune response by Tollo. (A) Dorsal view of *Ecc*-infected larvae of the following genotypes: Control (*Drs-GFP;;Tollo^C5^/+)*, *Tollo^-^* (*Drs-GFP;;Tollo^C5^/Tollo^R5A^), UAS-Tollo^IR^ (Drs-GFP;Btl-Gal4;UAS-Tollo^IR^*), *Tollo^-^PGRP-LC^-^* (*Drs-GFP;;Tollo^C5^,PGRP-LC^DE12^/Tollo^R5A^,PGRP-LC^DE12^*), *imd^-^Tollo^-^* (*Drs-GFP; imd^1^/imd^1^;Tollo^C5^/Tollo^R5A^*). The enhanced *Drs-GFP* expression observed in *Tollo* mutants is abolished when in conjunction with *PGRP-LC* or *imd* mutations. Images were taken 24h after *Ecc* infection. VB: visceral branch, DT: dorsal trunk, FB: fat body. (B) Infection-triggered AMP expression is enhanced in *Tollo* mutant trachea derived from third instar larvae (L3). The mRNA level post-infection in control flies (*Tollo^C5^/+*) was set to 100, and values obtained with other genotypes were expressed as a percentage of this value. Each histogram corresponds to the mean value +/− SD of 3 experiments. Values indicated by identical symbols (* or **) are not significantly different (*P*>0.05) from one another. All other differences are statistically significant (*P*<0.05). (C) The enhanced *Drosomycin* mRNA induction in *Tollo* single mutants is lost when in conjunction with *PGRP-LC* or *imd* mutations. mRNA levels post-infection in control flies (*OregonR*) was set to 100, and values obtained with other genotypes were expressed as a percentage of this value. Each histogram corresponds to the mean value +/− SD of 3 experiments. Values indicated by identical symbols (*, ** or ***) are not significantly different (*P*>0.05) from one another. All other differences are statistically significant (*P*<0.05). (D) Dorsal view and confocal pictures of *Ecc*-infected larvae of the following genotypes: Control (*Tollo^C5^, Dpt-Cherry/+, Dpt-Cherry*) and *Tollo^-^* (*Tollo^C5^,Dpt-Cherry/Tollo^R5A^, Dpt-Cherry)*. Pv: pro-ventriculus, Vtr: posterior end of ventriculus, Post CC: posterior to copper cells, Post mg: posterior midgut. Scale bar for confocal pictures is 100 µm. (E, F) Systemic AMP expression after infection in larvae (E) or in adults (F). The mRNA level post-infection in control flies (*Tollo^C5^/+*) was set to 100, and values obtained with other genotypes were expressed as a percentage of this value. Each histogram corresponds to the mean value +/− SD of 3 experiments. Values indicated by identical symbols (* or **) are not significantly different (*P*>0.05) from one another. All other differences are statistically significant (*P*<0.05).

The rather restricted expression pattern of *Tollo* mRNA in the trachea and the apical subcellular distribution of Tollo protein prompted us to investigate its putative function in the immune response. For that purpose, we used two previously characterized hypomorphic alleles (*Tollo^145^* and *Tollo^R5A^*) together with a complete loss-of-function allele (*Tollo^C5^*) that we generated by P-element mediated homologous recombination [31]–[32] (Figure S2A). All *Tollo* mutants were viable with no obvious developmental defects and gave rise to phenotypically normal pharate adults indicating that Tollo has no essential role in *Drosophila* development. We tested the ability of these *Tollo* mutant larvae to mount an immune response. In the absence of infection, approximately 5% of wild-type larvae showed *Drs-GFP* expression in VB and/or PS (Figure S3). Similar Figures were obtained with *Tollo* mutants suggesting that Tollo is not required to set up the basal level of AMP production in the absence of infection (Figure S3). After bacterial infection, however, the immune response was much stronger in *Tollo* mutants than in wild-type sibling larvae (Figure 4A and 4B and Figure S3). While we could identify the three previously described classes of *Drs-GFP* positive larvae in both control and *Tollo* mutants, the relative proportion of these was significantly different between genotypes (Figure S3). The percentage of larvae showing no GFP expression was reduced to 5–10% in *Tollo* mutant (compared to 40% in controls), whereas Class III larvae, which represented 15% of controls, reached up to 50% in the *Tollo* mutant larvae (Figure S3). Similar results were obtained with three independent *Tollo* alleles and in trans-heterozygous allelic combination demonstrating that this phenotype was, indeed, due to *Tollo* inactivation and not to other mutations on the chromosome (data not shown). The effects were not only qualitative but also quantitative. In most infected *Tollo* mutant larvae, *Drs-GFP* expression was intense, whereas it was rarely the case in controls (Figure 4A). q-RT-PCR experiments indicate that *Drosomycin*, *Drosocin* and *Attacin* mRNA levels were respectively increased by 6, 7 and 2.6 fold after infection in *Tollo* mutants compared to wild-type trachea (Figure 4B). To ensure that this effect was indeed a consequence of *Tollo* inactivation in the tracheal network itself, we combined the *Btl-Gal4* driver with a *UAS-Tollo^IR^* RNA interference construct. As shown in Figure 4A and Figure S3, larvae, in which *Tollo* was eliminated specifically in trachea, also showed increased *Drs-GFP* expression in this tissue after infection.

In addition to regulating *Drosomycin* expression in the trachea, the IMD pathway also controls *Diptericin* transcription locally in the gut and systemically in the fat body [13], [26]. To test whether Tollo acts as a general negative regulator of IMD-dependent mechanisms in other immune tissues, we analyzed the effects of inactivating *Tollo* on IMD pathway activation in the gut and the fat body. Using the *Dipt-Cherry* reporter construct for the larval stage (Figure 4D) and q-RT-PCR for both larval and adult stages (Figure 4E and 4F), we showed that *Tollo* was not implicated in IMD negative regulation in either tissue. This was the case for both immune responses induced by septic injury or by oral ingestion. In addition, we showed that *Tollo* mutants were unaffected in their ability to activate the Toll pathway during a Gram-positive bacteria-mediated systemic immune response (Figure 4F). Altogether these results demonstrate that the Tollo receptor is specifically required to dampen IMD pathway-dependent responses in the tracheal network after infection.

### AMP over-production in Tollo mutants is not secondary to trachea defects

**Figure 5:**
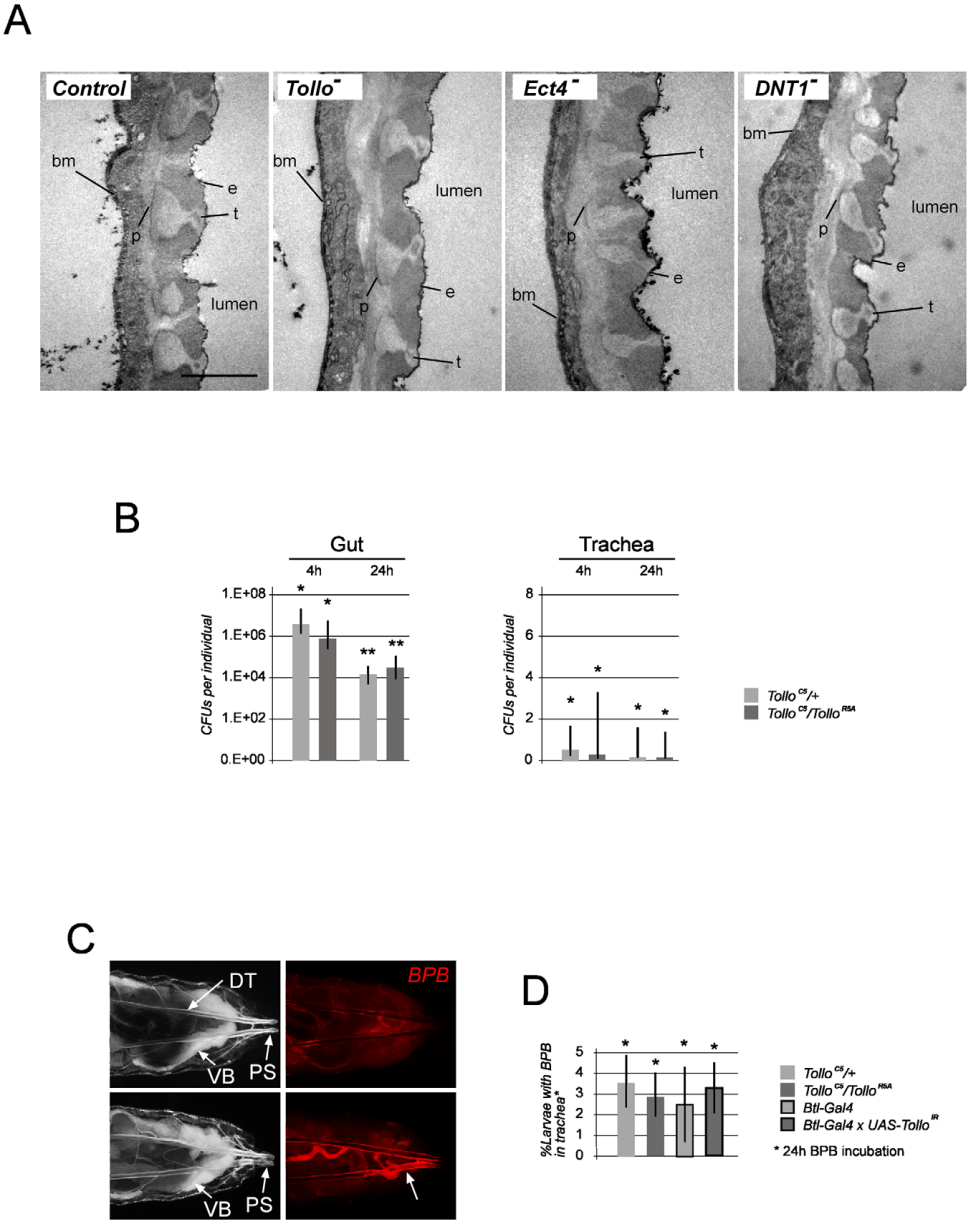
Tracheal morphology, putative immune elicitors and fluid penetration in trachea are not affected by *Tollo* mutations. (A) Electron microscopic pictures of tracheal transversal sections of control (*Oregon R*), *Tollo* mutant (*Tollo^C5^/Tollo^R5A^*), *Ect4* mutant (*Ect4^EY04273^/Df(3L)ED4408*) or *DNT1* mutant (*DNT1^41^/DNT1^41^*) third instar larvae. No obvious morphological differences could be observed between control and mutant trachea. (e) epicuticle, (t) taenidium, (p) procuticle and (bm) basement membrane. Scale bar is 2 µm. (B) Midgut and tracheal load of *Ecc-GFP* in control (*Tollo^C5^/+*) and *Tollo* mutant (*Tollo^C5^/Tollo^R5A^*) third instar larvae, 4h and 24 h post-infection. Note that bacterial load for *Ecc-GFP* is much weaker in trachea when compare to midgut. Colony forming units (CFUs) per tissue are shown for each condition. n = 6 in triplicates. (C) Example of control larvae (*Btl-Gal4*) without (top panels) or with (bottom panels) red fluorescent BPB in trachea (arrow). Dorsal views of third instar larvae are shown. Pictures were taken 24 h after Bromophenol Blue (BPB) incubation. (D) Quantification of (BPB) entry in trachea of non-infected larvae. BPB entry is rare (around 5%) and not affected by *Tollo* mutations (*Tollo^C5^/Tollo^R5A^*), *Tollo* RNAi (*Btl-Gal4;UAS-Tollo^IR^*) or infection (data not shown). Each histogram corresponds to the mean value +/− SD of 3 experiments. A total number of 90 larvae were counted for each experiment. Values indicated by identical symbols (*) are not significantly different (*P*>0.05) from one another.

Since AMPs are induced upon cellular stress, we tested whether *Drosomycin* expression in *Tollo* mutants was a secondary consequence of a possible implication of *Tollo* in tracheal formation. The following reasons led us to believe that it was not the case. 1) *Tollo* mutant embryos gave rise to viable adults, suggesting that *Tollo* mutant trachea are fully functional in larvae and adults. 2) Tracheal cell morphology of *Tollo* and control larvae appeared similar when observed under transmission electron microscopy (Figure 5A). 3) No constitutive AMP transcription was detected in non-infected *Tollo* mutant larvae (Figure 4B and Figure S3). We then wondered whether *Drosomycin* over-activation could be linked to the presence of higher levels of potential immune elicitors in *Tollo* mutant and RNAi trachea. This could be due to the presence of higher bacterial load in the trachea. However, as shown in Figure 5B and Figure S4B, the number of *Ecc-GFP* in *Tollo* mutant trachea was identical in *Tollo* mutants and in controls. Alternatively, *Tollo* mutant trachea could be more permeable to contaminated external fluid. To test this hypothesis, wild type and *Tollo* mutant larvae were incubated in the presence of a fluorescent dye, bromophenol blue. External fluid penetration inside the trachea lumen was not different in wild-type and *Tollo* mutant larvae (Figure 5C and 5D). This indicates that over-activation of *Drosomycin* in both *Tollo* mutant and RNAi trachea cannot be attributed to an increase in fluid penetration, and therefore putative immune elicitor load within the tracheal lumen. Altogether, these results demonstrate that the infection-dependent *Drosomycin* over-activation observed in *Tollo* mutant, is not secondary to defective trachea but rather suggests a direct implication of the Tollo protein in the regulation of IMD-dependent *Drosomycin* expression.

### Ect4/SARM and DNT1/Spätzle 2 loss-of-function mutants phenocopy Tollo mutants

**Figure 6:**
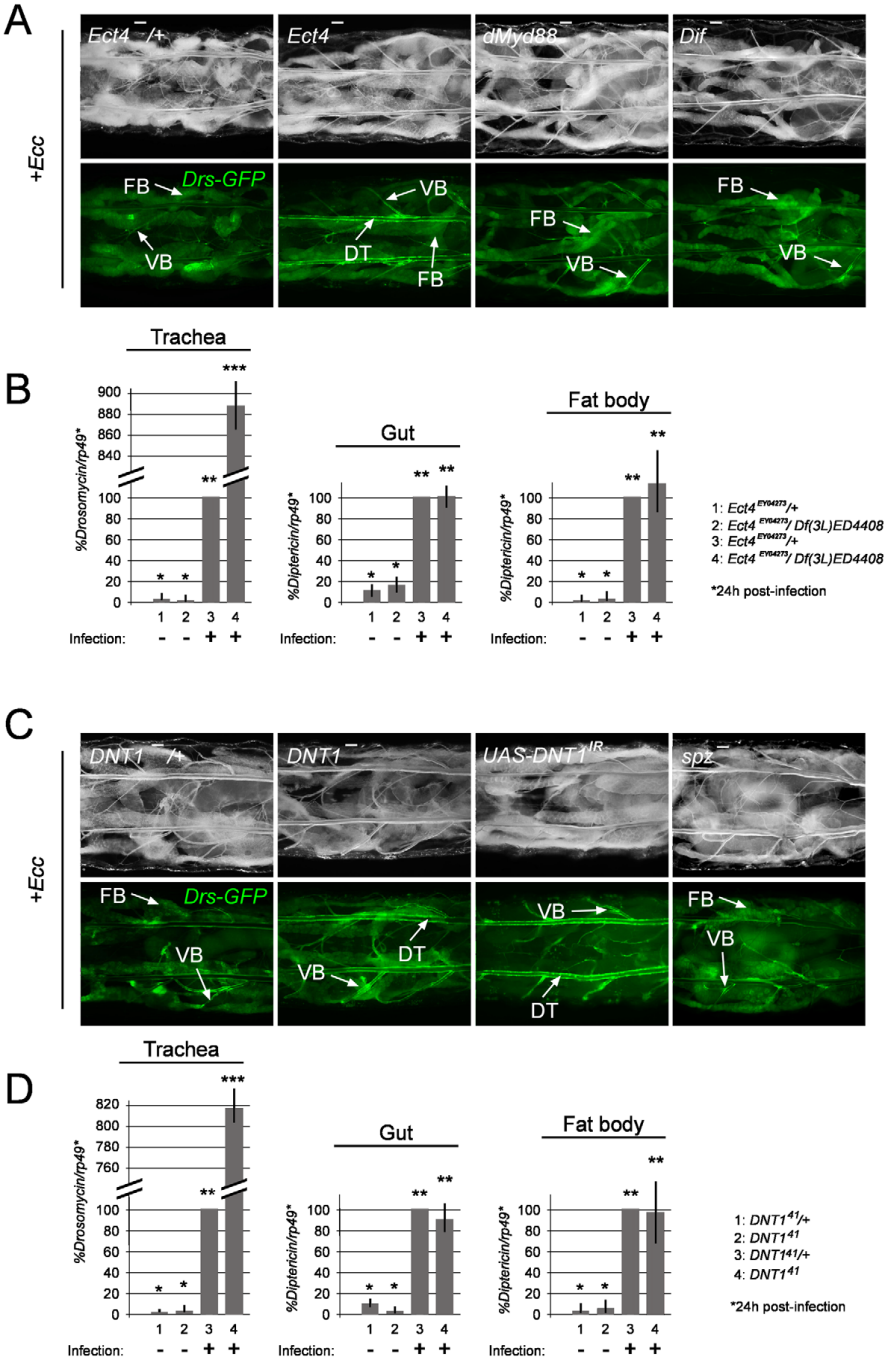
The TIR domain-containing protein Ect4/SARM and the cytokine DNT1/Spz2 negatively regulate tracheal immune response. (A) Dorsal view of *Ecc-*infected larvae of the following genotypes. *Ect4^-^*/+ (*Drs-GFP;;Ect4^EY04273^/+)*, *Ect4^-^ (Drs-GFP;;Ect4^EY04273^/Df(3L)ED4408*), *dMyd88^-^* (*Drs-GFP; dMyd88^c03881^/dMyd88^c03881^*) and *Dif- (Drs-GFP;Dif^1^/Dif^1^*). (B) Infection-triggered AMPs expression is enhanced in *Ect4* mutant trachea, but not in gut or fat body. mRNA levels post-infection in control flies (*Ect4^EY04273^/+*) was set to 100, and values obtained with other genotypes were expressed as a percentage of this value. Each histogram corresponds to the mean value +/− SD of 3 experiments. Values indicated by identical symbols (* or **) are not significantly different (*P*>0.05) from one another. All other differences are statistically significant (*P*<0.05). (C) *DNT1-/+*, (*Drs-GFP;;DNT1^41^/+*), *DNT1^-^*, (*Drs-GFP;;DNT1^41^/DNT1^41^*), *UAS-DNT1^IR^ (Drs-GFP*; *Btl-Gal4; UAS-DNT1^IR^)* and *spz^-^* (*Drs-GFP;; spz^rm7^/ spz^rm7^*). VB: visceral branch, DT: dorsal trunk, FB: fat body. Pictures were taken 24h after infection. (D) Infection-triggered AMPs expression is enhanced in *DNT1* mutant trachea, but not in gut or fat body. The mRNA level post-infection in control flies (*DNT1^41^/+*) was set to 100, and values obtained with other genotypes were expressed as a percentage of this value. Each histogram corresponds to the mean value +/− SD of 3 experiments. Values indicated by identical symbols (* or **) are not significantly different (*P*>0.05) from one another. All other differences are statistically significant (*P*<0.05).

We then tried to identify the intra- and extra-cellular components that may mediate *Tollo* signalling in trachea. The *Drosophila* Toll-1 receptor and vertebrate TLR functions have all been shown to be mediated by TIR domain-containing proteins, respectively, the *Drosophila* dMyd88 and the mammalian Myd88, TRIF, SARM, TRAM and MAL [33]–[35]. However, the *Drosophila* proteome contains two TIR domain proteins, Ect4/SARM the *Drosophila* ortholog of vertebrate SARM and dMyd88, the latter mediating the Toll signalling during dorso-ventral axis specification and immune response [36]–[38]. In order to test whether Tollo acts through a TIR domain-containing protein, we analyzed the tracheal immune response of *dMyd88* and *Ect4/SARM* mutants. We showed that larvae carrying *Ect4/SARM* mutations (Figure S2B) display a strong over-activation of *Drosomycin* expression (visualized with the *Drs-GFP* reporter transgene and quantified by q-RT-PCR) upon infection, phenotype that was not observed in *dMyd88* mutant larvae (Figure 6A and 6B and Figure S5A). This suggests that Ect4/SARM is the *bone fide* TIR domain adaptor transducing *Tollo* signalling in the tracheal immune response. Whereas TLRs function as Pattern Recognition Receptors by directly binding to microbial motifs, previous work has shown than the *Drosophila* Toll-1 receptor is activated during both embryonic dorso-ventral axis specification and immune response by its ligand Spätzle [39]–[40]. Since *spz* mutant larvae did not present higher activation of *Drs-GFP* in the respiratory tract after infection, we believe that Spz is not a functional ligand for *Tollo* (Figure 6C). Since *Spz*-like genes are present in the fly genome, we screened them for a *Tollo*-like phenotype. We observed that removing Spz2 (known as DNT1) function in trachea phenocopies *Tollo* mutant as far as *Drosomycin* over-activation is concerned (Figure 6C and 6D and Figure S5B). This suggests that DNT1 could be the, or one of the ligand(s), responsible for Tollo activity in the tracheal immune response. Similarly to Tollo, q-RT-PCR data indicate that Ect4 and DNT1 are specifically acting in the tracheal epithelium and do not contribute to IMD pathway regulation in the gut and in the fat body (Figure 6B and 6D).

### Tollo is hypostatic to IMD in the trachea

Taking into account the above results, it appears that the function of *Tollo* is specifically to down-regulate the IMD pathway in the tracheal cells following infection. In order to genetically place Tollo with respect to known IMD pathway components, we performed epistatic experiments. We showed that the *Tollo* mutant phenotype requires functional PGRP-LC receptor and intracytoplasmic adaptor IMD, since double mutant *Tollo^-^*, *PGRP-LC^-^* and *imd ^-^; Tollo^-^* trachea did not show any signs of *Drs-GFP* activation after infection (Figure 4A). This epistatic relationship was confirmed by q-RT-PCR on *Drosomycin* mRNA (Figure 4C). In genetic terms, *Tollo* is hypostatic, or acts in parallel to *PGRP-LC* and *imd*. Consistently, Relish nuclear translocation monitored with an anti-Relish antibody was higher in *Tollo* RNAi-infected tracheal cells than in controls (Figure S6). These results suggest that Tollo is not directly involved in IMD pathway activation *per se* but that, in its absence, IMD pathway activation is more efficient upon infection.

## Discussion

Epithelial responses are first and foremost local responses to prevent the epithelium from unnecessary immune reactions. Since the recognition steps in *Drosophila* respiratory epithelia involve the transmembrane receptor PGRP-LC and occur within the extracellular space, it is expected that molecular mechanisms must be at work to prevent constitutive or excessive immune response in this tissue, particularly essential for animal growth and viability. In this report, we present data demonstrating that the transmembrane receptor Tollo is part of a signalling network, whose function is to specifically down-regulate AMP production in the trachea. We show that Tollo antagonizes IMD pathway activation in the respiratory epithelium, and that DNT1/Spz2 and Ect4/SARM are putative Tollo ligand and transducer, respectively, in this process. Our data demonstrate that, in addition to the family founder Toll-1, another member of the Leucine-Rich-Repeats family of Toll proteins, is regulating the *Drosophila* innate immune response. Although it has been abundantly documented that every single mammalian TLR has an immune function [4], the putative implication of Toll family members, other than Toll-1 itself, in the *Drosophila* immune response has been a subject of controversy [41]. Data showing that *Drosophila* Toll-9 over-expression was sufficient to induce AMPs expression *in vivo* has prompted the idea that Toll-9 could maintain significant levels of anti-microbial molecules, thus providing basal protection against microbes [42]. However, our recent analysis of a complete Toll-9 loss-of-function allele has shown that this receptor is neither implicated in basal anti-microbial response nor required to mount an immune response to bacterial infection [43]. The present data are also fully consistent with a recent report showing that Toll-6, Toll-7 and Toll-8 are not implicated in systemic AMP production in flies [44], and demonstrate that a Toll family member, Tollo, is a negative regulator of local airway epithelial immune response upon bacterial infection. In contrast to Toll-1, whose activation is inducible in the fat body, Tollo pathway activation seems to be constitutive in the trachea. Despite these differences, both receptors use a member of the Spz family as ligand. Interestingly, sequence similarities, intron's size and conservation of key structural residues, indicate that Spz2/DNT1 is phylogenetically the closest family member to the Toll ligand Spz [45]. Furthermore, both Spz and Spz2/DNT1 have been shown to have neurotrophic functions in flies [46]. It would be of great interest to test whether Tollo also mediates Spz2 function in the nervous system.

Both during embryonic development and immune response, Spz is activated by proteolytic cleavage [10], [47]-[48]. This step depends upon the Easter protease that is implicated in D/V axis specification and on SPE for Toll pathway activation by microbes. Since Spz orthologs are also produced as longer precursors, they are likely to be activated by proteolysis. The fact that *Tollo* and *Spz2* loss-of-function phenotypes correspond to excessive AMP production, suggests that in wild-type conditions, the Tollo pathway is constitutively activated by an active form of the Spz2 ligand. This situation is reminiscent to that observed in the embryonic ventral follicle cells, in which a Pipe-mediated signal induces a constitutive activation of the Easter cascade leading to Spz cleavage, Toll activation and, in turn, ventral fate acquisition [49]. It should be noted that Easter and one Pipe isoform are very strongly expressed in the trachea cells (Flyatlas), and are candidate proteins in mediating Tollo activity in the respiratory epithelia.

The fact that *Ect4,* but not *dMyd88* mutant, loss-of-function mutant phenocopies *Tollo* mutant suggest that Ect4 could be the TIR domain adaptor transducing Tollo signal in the tracheal cells. Alternatively, Ect4/SARM could mediate Tollo function by interfering with IMD pathway signalling. In mammals, SARM is under the transcriptional control of TLR and negatively regulates TLR3 signalling by directly interfering with the association between the RHIM domain-containing proteins TRIF and RIP [50]. Since PGRP-LC contains a RHIM domain as TRIF, and IMD is the *Drosophila* counterpart of RIP, one can envisage that *Drosophila* SARM could act by interfering with the PGRP-LC/IMD association required for IMD pathway signalling. Similarly to its function as a negative regulator in fly immunity, SARM is the only TIR domain-containing adaptor that acts as a suppressor of TLR signalling [36], [50].

One obvious question relates to the mode of action of Tollo on IMD pathway downregulation. Two mechanisms have been recently described that result in the down-regulation of the IMD pathway. The first one regulates PGRP-LC membrane localization, and is dependent on the PIRK protein [51]–[53]. Upon infection, the intracellular PIRK protein is up-regulated and, in turn, represses PGRP-LC plasma membrane localization leading to the shutdown of the IMD signalling [53]. In infected *pirk* mutants, IMD-dependent AMPs are overproduced in both the gut and the fat body. In our conditions, however, inactivation of PIRK specifically in the trachea did not influence *Drosomycin* activation in trachea (Figure S7A). To verify whether *Tollo* is acting via a mechanism similar to PIRK, we looked at PGRP-LC membrane localization using a *UAS-PGRP-LC::GFP* construct. PGRP-LC membrane localization was identical in wild-type and *Tollo* mutant tracheal cells (Figure S7B). The second mechanism that modulates IMD activation, acts directly on the promoters of IMD target genes. Ha *et al.* (2005) have shown that the Caudal transcription factor sits on some of the IMD target promoters preventing their activation by Relish [54]. We thus tested the putative implication of Caudal in Tollo signalling by using *Drs-GFP* reporter transgenes containing either wild-type Caudal Responsive Elements (CDREs) or mutated versions unresponsive to Caudal activity [55]. Upon infection, *Drs-GFP* with mutated CDREs was activated in fat body but not in gut or trachea (Figure S7C). In conclusion, Caudal acts as a transcriptional activator, rather than a repressor, for the *Drs-GFP* reporter in trachea. These results indicate that Tollo does not regulate the IMD pathway via PGRP-LC membrane localization or through promoter targeting of Caudal. One challenging task for the future will be to identify the mechanism used by Tollo to counter-balance tracheal PGRP-LC activation. It has been reported that the loss of *Tollo* function in ectodermal cells during embryogenesis alters glycosylation in nearby differentiating neurons [31], [56]–[57]. Since the pattern of oligosaccharides expressed in a cell can influence its interactions with others and with pathogens, Tollo could function by modifying glycosylation pattern in response to microbes. It could be envisaged that Tollo mediates PGRP-LC glycosylation, and thereby reduces its ability to respond to bacterial elicitors. Further work will be required to address the above hypothesis, whereby Tollo activity and glycosylation modification could be linked in order to regulate the IMD pathway activation in trachea.

## Material and Methods

### Bacterial strains

The following microorganisms were used: *Erwinia carotovora carotovora 15 2141 (Ecc), Erwinia carotovora carotovora 15 pOM1-GFP spectinomycin^R^ (Ecc-GFP), Escherichia coli 1106 (E.coli)* and *Micrococcus luteus* CIPA270 *(M. luteus)*.

### Bacterial load analysis

Bacterial load of surface sterilized individuals was quantified by plating appropriate serial dilutions of lysates obtained from 6 dissected guts or trachea (from larvae) on nutrient agar plates (Luria Bertani + spectinomycin 100 µg/ml). Biological triplicates were collected for each experimental condition at 4h and 24h after *Ecc-GFP* infection. Homogenization of tissues was performed using the Precellys 24 tissue homogenizer (Bertin technologies, France) and 0,75-1mm glass beads in 500 µL of LB + spectinomycin.

### Drosophila melanogaster strains and maintenance

*PGRP-LC^DE12^* is a complete deletion of the *PGRP-LC* locus [16]. Flies carrying this mutation are unable to activate the IMD pathway. *spz^rm7^ i*s a null allele which prevents Toll pathway activation [5]. *yw, Drs-GFP* [*27*], *Dpt-Cherry* [58], *Tollo^C5^* (this work), *Tollo^R5A^* [32], *Tollo^145^* [59], *UAS-Tollo^IR^* (VDRC #9431), *UAS-Tollo::Myc* [31], *DNT1^41^* [46], *UAS-spz2^IR^* (VDRC #26115), *Ect4^EY04273^* BL#15733, *Df(3L)ED4408* BL#8065, *Tl^3^* BL#3238 (a dominant gain-of-function allele of Tl, *Btl-Gal4* BL#8807, *UAS-myrRFP* BL#7118, *act>CD2>Gal4* BL#4780, *cad-EGFP* BL#30875, *Vkg-GFP* (a gift from Michel Sémériva), *hs-Gal4* BL#2077, *Relish^E20^* [60], *imd^1^* [5], *UAS-spz act* [30], *dMyd88^c03881^* [34], *UAS-PGRP-LC::GFP* (a gift from François Leulier) and *Dif^1^* [61]. Generation of the *Tollo^C5^* allele was performed as described in [62] using the two following inserted elements: d01565 and PBacf05248 [63]. Complete deletion of the *Tollo* gene was confirmed by sequencing genomic DNA extracted from *Tollo^C5^* mutants (molecular details upon request). Fly stocks were raised on standard cornmeal-agar medium at 25°C.

### Natural infection of larvae and adults

Cells from overnight bacterial cultures were recovered by centrifugation at 4,000 g for 10 min at 4°C. The supernatant was discarded and the pellet was resuspended in fresh LB media. Cell suspensions were serially diluted in PBS, and the concentration of cells was determined by optical-density (OD) measurement. 200 µl of an overnight bacterial culture of *Ecc* (OD = 200) were directly added on top of feeding third instar larvae into a standard cornmeal-agar medium at 25°C. A similar volume of LB broth was used in control experiments. Larvae were monitored for *Drosomycin* and *Diptericin* transcription by fluorescence analysis using *Drs-GFP* and *Dpt-cherry* reporters respectively, and by qRT-PCR, 24h after infection. Septic injuries were performed by pricking adult males with a thin needle contaminated with *M. luteus* or *E. coli*. 200 µl of Bromophenol Blue (SIGMA # B8026) at 10 g/l were directly added on top of feeding third instar larvae.

### Flip-out clones and MARCM

For *Drs-GFP* study, *Drs-GFP;UAS-myrRFP;act>CD2>Gal4* females were crossed to either *ywhsflp;;UAS-IMD* or to *ywhsflp;; UAS-PGRP-LCa* males. For *Dpt-Cherry* study, *ywhsflp; UAS-GFP; act>CD2>Gal4* females were crossed to *Dpt-Cherry; UAS-IMD* or to *Dpt-Cherry, UAS-PGRP-LCa* males. In both cases, larvae of the progeny were heat shocked at early-mid L3 stage (72h-96h after egg deposition, AED) and observed 24 h later. Generation of MARCM clones in trachea was performed by crossing MARCM virgin females of genotype *ywhsflp;; Tub-Gal80 FRT2A* en masse to the *Drs-GFP; Blt-Gal4, UAS-myrRFP; PGRP-LCD^E12^ FRT2A* line. Resulting embryos were submitted to a heat shock 4–6 hr AED for 1 hr at 38°C in a circulating water bath, and kept at 25°C until larvae reached early-mid third instar (72h-96h AED), when they were infected by *Ecc* and observed 24 h later.

### Immunostaining on larvae

Larval tissue were dissected in PBS and fixed for 20 min in 4% paraformaldehyde on ice. After several rinses in PBT (PBS + 0.1% Triton X-100), they were blocked for 1 hr in PBT-3% BSA at 4°C and then incubated with antibody at the appropriate dilution in PBT-BSA 3% overnight at 4°C. Primary antibodies were: rabbit Anti-Relish (1∶500) or Mouse Anti-Myc (9E10 Santa Cruz at 1∶ 500). Several washes in PBT were followed by a 2 hr incubation with secondary antibody at RT (Alexa Fluor 546 goat anti-rabbit IgG and Alexa Fluor 555 goat anti-mouse IgG diluted 1∶500, Molecular Probes), then 5 washes in PBT. The tissues were finally mounted in Vectashield (Vector Laboratories) fluorescent mounting medium, with DAPI. Images were captured with a LSM 510 Zeiss confocal microscope.

### Quantitative real-time PCR

Quantitative real-time PCR and SYBR Green analysis were performed as previously described [58]. Primer information can be obtained upon request. The amount of mRNA detected was normalized to control *rp49* mRNA values. Normalized data was used to quantify the relative levels of a given mRNA according to cycling threshold analysis (ΔCt).

### Electronic microscopy

For electron microscopic sections, third instar larvae trachea were dissected and fixed at RT in 4% PFA and 2% glutaraldehyde in 0.12 M sodium cacodylate buffer at pH 7.4 for 1 h. The trachea were then washed 3×10 min in 0.12 M sodium cacodylate buffer, post-fixed in 2% OsO4 in 0.12 M sodium cacodylate buffer for 1 h and washed again 3×10 min. Samples were subsequently dehydrated through series of ethanol gradients and infiltrated with propylene oxide, embedded in epoxy resin (Fluka, Sigma) and polymerized at 80°C. Ultrathin (80 nm) plastic sections were cut using a Leica UltraCut microtome with a diamond Diatome knife and post-stained with 2% uranyl acetate, followed by treatment with Reynolds'lead citrate, and stabilized for transmission EM by carbon coating. Examination was performed with a Zeiss Leo 912 microscope at 100 kV. Images were captured using a Gatan 792 Bioscan camera using Digital Micrograph software.

## Supporting Information

Figure S1 ***Ecc-*** **mediated** ***Drs-GFP*** **activation in the trachea is IMD-dependent and Toll-independent.** (A) Dorsal views of *Drs-GFP* larvae of the following genotypes: *UAS-IMD* (*Drs-GFP;hs-Gal4/+;UAS-imd/+)*, *UAS-PGRP-LCa* (*Drs-GFP;hs-Gal4/+;UAS-PGRP-LCa/+)*, *UAS-spz act* (*Drs-GFP;hs-Gal4/UAS-spz act)*, *Tl^3^ (Drs-GFP;Tl^3^/+)*, *PGRP-LC^DE12^* (*Drs-GFP;; PGRP-LC^DE12^/PGRP-LC^DE12^*), *imd^1^ (Drs-GFP;imd^1^/imd^1^) and Relish^E20^ (Drs-GFP; Relish^E20^/Relish^E20^*). In non-infected larvae, gain-of-function mutations of IMD pathway components, but not of Toll pathway components, are sufficient to promote intense expression of *Drs-GFP* in trachea. Upon *Ecc* infection, *Drs-GFP* expression is lost in *PGRP-LC*, *imd* or *Relish* mutants. Images were taken 24h after heat-shock or *Ecc* infection. PS: posterior spiracles, VB: visceral branch, DT: dorsal trunk, FB: fat body. (B) Quantification of *Drs-GFP* expressing larvae is displayed as histograms. Statistics apply for the “no signal” and the Class III categories only. Each histogram corresponds to the mean value of 5 experiments. A total number of 80 larvae were counted for each experiment. Values indicated by identical symbols (*, ** or ***) are not significantly different (*P*>0.05) from one another. All other differences are statistically significant (*P*<0.05).
(TIF)

Figure S2 **Down regulation of** ***Tollo*** **mRNA in** ***Tollo*** **mutants and** ***Tollo*** **RNAi larvae and down regulation of** ***Ect4*** **mRNA in** ***Ect4*** **mutant.** (A) *Tollo* mRNA detection by q-RT-PCR is shown for total third instar larvae or dissected trachea. *Tollo* mRNA measured in wild-type (*OregonR*) larval tissue was set to 100, and values obtained with other tissues were expressed as a percentage of this value. Histograms correspond to the mean +/- SD of 3 experiments. Values indicated by identical symbols (* or **) are not significantly different (*P*>0.05) from one another. All other differences are statistically significant (*P*<0.05). (B) *Ect4* mRNA detection by q-RT-PCR is shown for dissected trachea. *Ect4* mRNA measured in wild-type (*OregonR*) larval tissue was set to 100, and values obtained with other tissues were expressed as a percentage of this value. Histograms correspond to the mean +/- SD of 3 experiments. Values indicated by different symbols (* and **) are significantly different from one another (*P*<0.05).
(EPS)

Figure S3 **Negative regulation of tracheal immune response by Tollo.** Quantification of *Drs-GFP* expressing larvae in various genotypes. Tracheal expression of *Drs-GFP* in *Ecc*-infected larvae is enhanced by either *Tollo* mutations or *Tollo* RNAi-mediated inactivation. Statistics apply for the “no signal” and the class III categories only. Each histogram corresponds to the mean value of 8 experiments. A total number of 120 larvae were counted for each experiment. Values indicated by identical symbols (*, ** or ***) are not significantly different (*P*>0.05) from one another. All other differences are statistically significant (*P*<0.05).
(EPS)

Figure S4 **Bacterial penetration into larval trachea upon** ***Ecc*** **infection.** (A) *Ecc-GFP* localization in trachea of wild-type larvae. *Ecc-GFP* can be found either in posterior spiracle (PS), visceral branches (VB) or dorsal trunk (DT). Pictures were taken 24h after infection. (B) Histograms show quantification of larvae with *Ecc-GFP* positive trachea in control (*Tollo^C5^/+* and *Btl-Gal4*) or *Tollo* mutants (*Tollo^C5^/Tollo^R5A^*) and *Tollo* RNAi (*Btl-Gal4; UAS-Tollo^IR^*) third instar larvae. Each histogram corresponds to the mean value of 5 experiments. A total number of 100 larvae were counted for each experiment. Values indicated by identical symbols (*) are not significantly different (*P*>0.05) from one another.
(EPS)

Figure S5 **The TIR domain-containing protein Ect4 (SARM) and the cytokine Spz2 (DNT1) negatively regulate tracheal immune response.** (A, B) Quantification of *Drs-GFP* expressing larvae in various genotypes. Statistics apply for the “no signal” and the class III categories only. Each histogram corresponds to the mean value of 3 experiments. A total number of 80 larvae were counted for each experiment. Values indicated by identical symbols (*, ** or ***) are not significantly different (*P*>0.05) from one another. All other differences are statistically significant (*P*<0.05).
(EPS)

Figure S6 **Nuclear localization of Relish is increased in** ***Tollo*** **RNAi trachea.** Confocal images representative of control or *Tollo* RNAi trachea from *Ecc-*infected larvae and stained with anti-Relish antibody (Red). Nuclei are stained with Dapi (blue). Scale bar is 50 µm.
(EPS)

Figure S7 **Negative regulation by Tollo does not involve PIRK,PGRP-LC membrane localization or Caudal Responsive Elements.** (A) Quantification of *Drs-GFP* expressing larvae in various genotypes. Tracheal expression of *Drs-GFP* in *Ecc*-infected larvae is unaffected by *pirk* RNAi-mediated inactivation. Statistics apply for the “no signal” and the class III categories only. Each histogram corresponds to the mean value of 8 experiments. A total number of 80 larvae were counted for each experiment. Values indicated by identical symbols (* or **) are not significantly different (*P*>0.05) from one another. All other differences are statistically significant (*P*<0.05). (B) Tollo is not affecting PGRP-LC::GFP membrane localization in trachea. Confocal images representative of control or *Tollo* mutant trachea expressing a *PGRP-LC::GFP* fusion protein, in third instar larvae. The apical localization of PGRP-LC::GFP is un-affected by *Tollo* mutations. The following genotypes are shown: *Tub-Gal4, Tub-Gal80^ts^/+; Tollo^C5^/+* (control) and *Tub-Gal4, Tub-Gal80^ts^/+; Tollo^C5^/Tollo^R5A^*. A total number of 30 trachea were observed for each genotype. Images were taken 24h after incubation at 29°C. (C) Image representative of *Ecc-*infected *Drs(CDREmut)-GFP* larvae is shown with no GFP signal visible in trachea (lower panel). The CDREs of *Drosomycin* promoter are required for tracheal expression upon *Ecc* infection. A total number of 50 larvae were analyzed. Images were taken 24h after *Ecc* infection.
(EPS)
