# Supplementary figures and images for: Toll-8/Tollo Negatively Regulates Antimicrobial Response in the Drosophila Respiratory Epithelium
Source: PLoS Pathog. 2011 Oct 13;7(10):e1002319. doi: 10.1371/journal.ppat.1002319 (PMC3192845; doi:10.1371/journal.ppat.1002319)

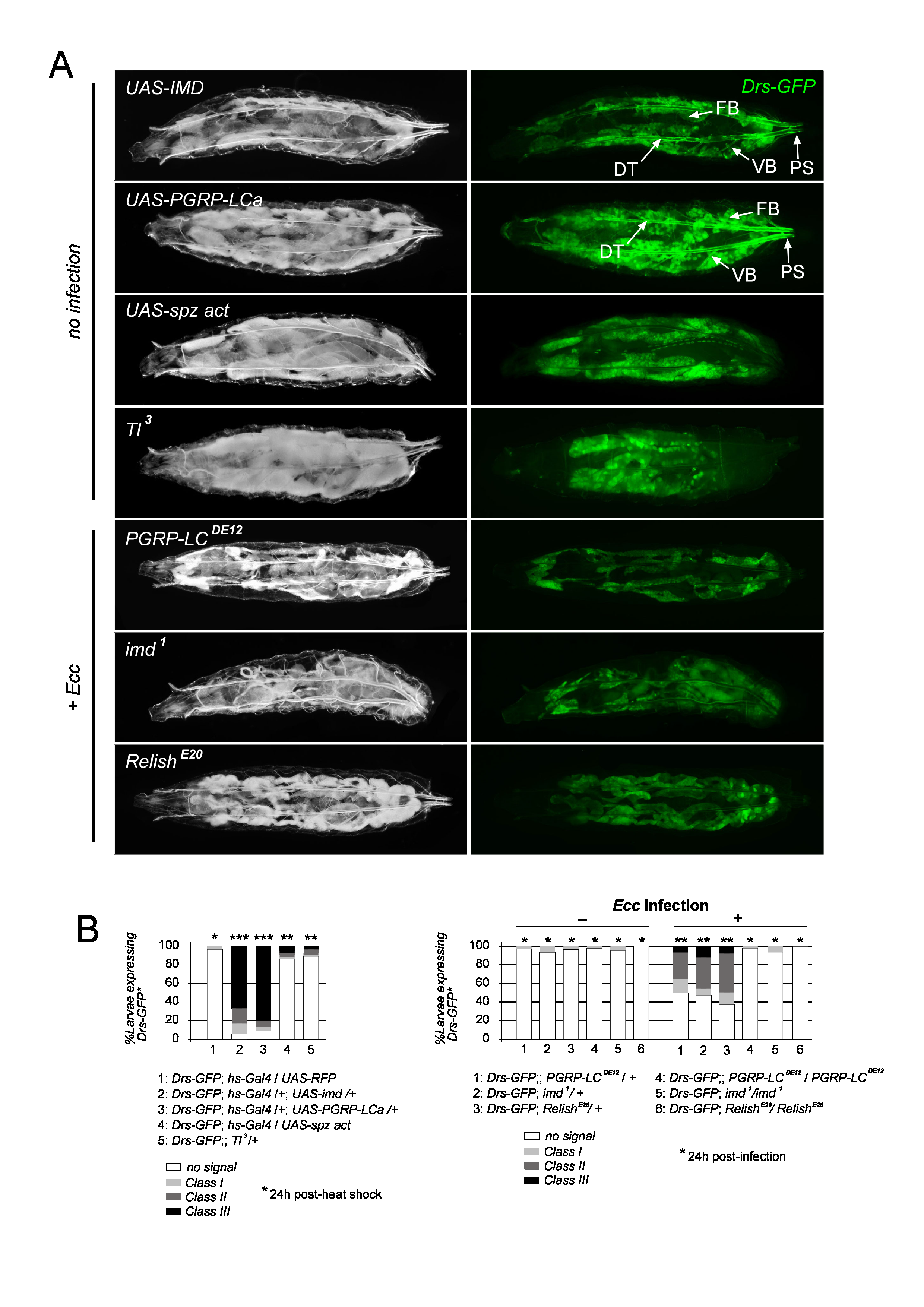

Supplement: Figure S1 — Ecc- mediated Drs-GFP activation in the trachea is IMD-dependent and Toll-independent. (A) Dorsal views of Drs-GFP larvae of the following genotypes: UAS-IMD (Drs-GFP;hs-Gal4/+;UAS-imd/+), UAS-PGRP-LCa (Drs-GFP;hs-Gal4/+;UAS-PGRP-LCa/+), UAS-spz act (Drs-GFP;hs-Gal4/UAS-spz act), Tl3 (Drs-GFP;Tl3/+), PGRP-LCDE12 (Drs-GFP;; PGRP-LCDE12/PGRP-LCDE12), imd1 (Drs-GFP;imd1/imd1) and RelishE20 (Drs-GFP; RelishE20/RelishE20). In non-infected larvae, gain-of-function mutations of IMD pathway components, but not of Toll pathway components, are sufficient to promote intense expression of Drs-GFP in trachea. Upon Ecc infection, Drs-GFP expression is lost in PGRP-LC, imd or Relish mutants. Images were taken 24h after heat-shock or Ecc infection. PS: posterior spiracles, VB: visceral branch, DT: dorsal trunk, FB: fat body. (B) Quantification of Drs-GFP expressing larvae is displayed as histograms. Statistics apply for the “no signal” and the Class III categories only. Each histogram corresponds to the mean value of 5 experiments. A total number of 80 larvae were counted for each experiment. Values indicated by identical symbols (*, ** or ***) are not significantly different (P>0.05) from one another. All other differences are statistically significant (P<0.05). (TIF) [file ppat.1002319.s001.tif]
